# Combining Qualitative and Quantitative Evidence to Determine Factors Leading to Late Presentation for Antiretroviral Therapy in Malawi

**DOI:** 10.1371/journal.pone.0027917

**Published:** 2011-11-16

**Authors:** Fiona R. Parrott, Charles Mwafulirwa, Bagrey Ngwira, Sothini Nkhwazi, Sian Floyd, Rein M. G. J. Houben, Judith R. Glynn, Amelia C. Crampin, Neil French

**Affiliations:** 1 Faculty of Epidemiology and Population Health, London School of Hygiene & Tropical Medicine, London, United Kingdom; 2 Karonga Prevention Study, Chilumba, Malawi; 3 Amsterdam Institute for Social Science Research (AISSR), University of Amsterdam, Amsterdam, The Netherlands; University of Cape Town, South Africa

## Abstract

**Background:**

Treatment seeking delays among people living with HIV have adverse consequences for outcome. Gender differences in treatment outcomes have been observed in sub-Saharan Africa.

**Objective:**

To better understand antiretroviral treatment (ART) seeking behaviour in HIV-infected adults in rural Malawi.

**Methods:**

Qualitative interviews with male and female participants in an ART cohort study at a treatment site in rural northern Malawi triangulated with analysis of baseline clinical and demographic data for 365 individuals attending sequentially for ART screening between January 2008 and September 2009.

**Results:**

43% of the cohort presented with late stage HIV disease classified as WHO stage 3/4. Respondents reported that women's frequency of testing, health awareness and commitment to children led to earlier ART uptake and that men's commitment to wider social networks of influence, masculine ideals of strength, and success with sexual and marital partners led them to refuse treatment until they were sick. Quantitative analysis of the screening cohort provided supporting evidence for these expressed views. Overall, male gender (adjusted OR 2.3, 95% CI1.3–3.9) and never being married (adjusted OR 4.1, 95% CI1.5–11.5) were risk factors for late presentation, whereas having ≥3 dependent children was associated with earlier presentation (adjusted OR 0.31, 95% CI0.15–0.63),compared to those with no dependent children.

**Conclusion:**

Gender-specific barriers and facilitators operate throughout the whole process of seeking care. Further efforts to enrol men into care earlier should focus on the masculine characteristics that they value, and the risks to these of severe health decline. Our results emphasise the value of exploring as well as identifying behavioural correlates of late presentation.

## Introduction

The roll-out of antiretroviral therapy (ART), free at the point of delivery began in Malawi in 2004 [1]. It has resulted in improved survival in the HIV-infected [2], [3] with measurable impact on adult mortality at the population level [4]. However many HIV-positive individuals continue to present for the first time with advanced HIV disease in Malawi [3], [5], and elsewhere in sub-Saharan Africa [6], [7], [8].

Patients with advanced disease are at risk of early death [7], [8], do not receive the maximum benefits of screening for tuberculosis, prophylactic drugs to reduce the incidence of opportunistic infections, or educational interventions, and put pressure on clinical resources [9], [10], [11]. Earlier presentation and treatment also reduces viral loads and risk of transmission to others [6], [12]. Understanding why people living with HIV postpone seeking treatment until severe health decline will facilitate development of strategies to improve efficiency of the ART programme.

In other studies, factors promoting delay include fear of HIV/AIDS stigma, side-effects, hunger and taking ART on an empty stomach, perceptions of illness susceptibility, and depression [13], [14], [15], [16], [17]; and program factors including overcrowding, overworked staff and poor linkage between services [14]. Seeing people becoming well, shifting community attitudes and support from family and spouse have been described as facilitators [13], [14]. Several studies from sub-Saharan Africa have shown that male gender, older age and socioeconomic factors, such as lack of secondary school education and unemployment, are associated with late presentation [6], [7].

Male gender and older age have been associated with early death on ART in Malawi [3]. Men's early mortality appears mainly to be due to presentation with more advanced HIV disease [8], [18], although compliance with therapy and other health facility related factors also contribute [3]. Men may be less likely to seek medical help in general [19], and particularly so in the context of HIV [19]. Men's position of responsibility within the family and the shame they feel when accessing ART have been described as barriers to seeking care [20], [21], [22]. Other barriers include high mobility occupations [23] and use of alcohol [21]. In contrast, maternal and child health policies which reinforce women's HIV testing and their role in children's health facilitate earlier attendance for women [24]. Finding ways to promote earlier access among men is a priority to improve programme outcomes [8].

Understanding the way health behaviours are influenced by constructions of masculinity and femininity [25], needs to be supported by an account of the relationship between behaviours and outcomes [26]. Qualitative studies are crucial to understanding what motivates or supports people to seek care [27]. Quantitative studies can assess the wider applicability of the findings and the strength of associations in the general population. However, studies using these complementary methods are rarely conducted.

Given the importance of timely initiation of ART and the need to promote earlier access, we carried out a mixed methods study in Malawi to explore the reasons why some people sought treatment before severe health decline and others only after delay, paying particular attention to the role of gender in shaping these experiences.

## Methods

### Ethics statement

Ethical approval was obtained from the National Health Sciences Research Council of Malawi (642) and the London School of Hygiene and Tropical Medicine Ethics Committee (5535). Written informed consent was obtained from all participants.

### Study Setting

The study was conducted in Karonga District, northern Malawi. The population are predominantly Tumbuka-speaking and Christian, in a subsistence economy of farmers and fishermen, with small businesses in the semi-urban trading centre and the lake-side port. Households have an average of 5 members, only 15% of households rely on salaried employment as their main source of income and few are supplied with electricity (3%) or piped water (16%) [28]. Almost three quarters of men complete primary education (standard 8) compared with less than two thirds of women [29].

Median age at first marriage is 18.5 years among women and 23.7 years among men [29]. Divorce rates are high [28] and remarriage of widowed or divorced women after age 35 is uncommon [30]. Polygyny increases with age reaching 20% in men from age 30–34 [28]. Descent is patrilineal and marital residence is usually with or near the husband's family. Women are expected to show respect to their husbands and in-laws but retain strong links with their natal family [31]. A high value is placed on parenthood. As mothers, women receive considerable respect and seniority increases as adult sons become heads of household.

The study is set in a rural clinic in the south of the district, within a population of 34,000 who are under continuous demographic surveillance [32]. ART was first available in the district in 2005 and in the study clinic in 2006. Two further clinics at local health centres opened in 2008. At the time of the study, under government guidelines, individuals were eligible for ART if in stage 3/4, or in stage 2 with CD4 cell count <250 cells/mm3. Before starting ART they must attend a group counselling session with their chosen treatment guardian, who may collect medicines on their behalf. Short-term ART for prevention of mother to child transmission was available via ANC clinics. HIV prevalence in this area was estimated at 11.4% in 2005–6; 13.7% among women and 9.1% among men [33]. The lower prevalence in men partially reflects older age at infection.

Residents of the demographic surveillance site (DSS) who presented for screening at the rural ART clinic from January 2008 were invited to participate in a cohort study of outcomes and prognostic indicators. Baseline recruitment procedures identified these individuals and their households in the database of the DSS population and recorded CD4 cell counts and WHO clinical stage. Late presentation was defined as those presenting in clinical stage 3 and 4.

### Qualitative data

A purposive sample of patients on ART, from strata defined by gender and grouped according to WHO clinical stage at presentation, were invited to participate in qualitative semi-structured interviews. These explored why and when they sought treatment and their perception of their health at the time. Interviews were conducted between July and September 2009 and were recorded in private, using the local language. The first interview was conducted in private at the hospital, followed by a lengthier visit to the participants' household. Timelines were created to help recall and explore testing as well as treatment-seeking events.

Interviews were translated into English by trained field workers. Open and axial coding of the translated text of interviews focused on factors that contributed to postponing seeking care until health decline, and on supports to seeking treatment, including the role of gender in shaping these experiences.

### Quantitative data

Adults who enrolled in the cohort between January 2008 and September 2009 were included. Individual and household characteristics were linked, when available from the demographic surveillance, from around the time the participant first screened at the ART clinic. Area of residence was characterised as roadside (≤1 km from the tarmac road) or remote rural; House construction was ranked according to the quality of building materials. The number of dependent children was defined as the number of biological children aged 14 years or below living in the household.

Univariable and multivariable logistic regression were carried out to identify factors associated with stage at presentation for screening. Analyses were done separately for men and women, and also combined after checking for interaction (using Likelihood ratio tests). Among married participants we compared those who attended before their spouses and those who attended second or together.

We employed a mixed method design (Qual - quant), using the quantitative component to enhance description and test conjectures from the qualitative component [34]. The quantitative analysis examined associations between socio-demographic characteristics and WHO clinical stage at first screening. The intention was to assess the generalisability of the qualitative findings and to establish whether expressed reasons for seeking or postponing care could be related to individual and household characteristics.

## Results

365 individuals enrolled in the clinical cohort during the study period. 36 individuals could not be linked to one or more socio-demographic variables as they were recent migrants. The majority were female (58%) and median age was 37years. 43% presented with late stage HIV disease, (WHO stage 3/4). Men were more likely to attend with late stage disease: 53% of men and 36% of women presented in stage 3/4 (OR 2.0, 95% CI1.3-3.1), with 17% of the men and 6% of the women in stage 4.

28 men and 32 women consented to in-depth interviews. A total of 120 interviews took place at the clinic and at their homes. 22 patients had died and 39 were no longer in the cohort study when participants were recruited for interview. Around 90% of those asked to participate consented, with refusals largely associated with bad weather at the time of recruitment. Table 1 gives the key demographic characteristics of the interview sample, alongside the cohort.

**Table 1:** Baseline description of ART cohort and interview sample.

Variable	ART cohortn = 365	Interview Samplen = 60
Gender (% male)	42%	47%
WHO stage 3/4	43%	50%
Age (median IQR)	37 (32–47)	37 (33–49)
Marriage		
Married	61%	50%
Divorced	16%	17%
Widowed	14%	16%
Never Married	8%	18%
Children		
None	37%	20%
1–2 children	42%	56%
3 or more children	21%	24%
Education		
None/Primary 1–5	17%	12%
Primary 6–8	51%	57%
Secondary/above	22%	31%
Occupation		
Farmer	64%	57%
Professional	5%	8%
Small Trader/Manual Labourer	13%	35%
Housing Quality		
Better quality	47%	44%
Poorer quality	53%	56%

### Reasons for late presentation

#### Attitudes towards testing, health education messages and beliefs about ART

Participants tested at government health centres, charitable clinics and a house-to-house survey. Almost a third of respondents re-tested at the study clinic's VCT facility after a positive test elsewhere; some by choice: “*I only believed the results after two tests*” (female, mid-thirties, stage 3). Women tested more frequently than men, particularly those of childbearing age due to ANC testing. Women were more likely to want to know their status, especially if they suspected a husband of having many girlfriends. Most men presenting with advanced disease described severe ill health before testing. By this point they no longer cared if they received a positive diagnosis: “*I was ready to accept any situation since the whole body was in serious pains and I felt I was already dead*” (male, early fifties, stage 4); “*I was ready for any outcome whether positive or negative. I didn’t care because of the [health] problems I faced...I never expected to be alive*” (male, early thirties, stage 4).

Men and women's attitudes to health promotion messages differed. Women regularly mentioned radio broadcasts, “*we like staying close to the radio and I find out that what is said is what I experience*” (female, late thirties, stage 2). Several men admitted ignoring such programmes, “*I could hear it [information on HIV] from the radio but I thought it was useless. I thought that’s what the government always does. If you are ignorant of something you ignore it*” (male, early fifties, stage 4). In general, men did not wish to appear weak, “*Men are proud, we think caring for our bodies is a waste of time*” (male, late thirties, stage 3). When men sought treatment, they spoke of being overpowered by HIV, “*You know what I have to do is accept the defeat*” (male, early thirties, stage 4).

All but one male respondent had known ART was freely available but both men and women described waiting “*to see for themselves the signs on their bodies*” (female, mid-thirties, stage 2). ART was believed to be a powerful medicine. Three people primarily delayed screening from fear. It’s potency also encouraged earlier treatment seeking, “*since the drug is strong, if it meets someone who is very weak it makes their health deteriorate, so it is better to go earlier*” (male, early forties, stage 2). Respondents had seen sick people recover: “*those people on ARVs look better than those who haven’t started taking them*” (female, mid-thirties, stage 2). Many drew hope from praying that ART would work for them.

Two women and a man said this was their second time to screen: they had lost touch with services after learning their ‘*defences*’ were too high and returned more than three years later with advanced disease.

#### ART clinic attendance and fear of losing respect

Most people believed that attending the clinic for treatment was tantamount to declaring their HIV status openly. A minority said they were better able to hide their status in their home village by starting treatment quickly, “*once you’ve started taking the medicine in good time, nobody will know you are taking ARVs*” (female, early thirties, stage 2). The costs of ‘becoming known’ differed in severity and kind. Older women were more likely to describe the shame of being thought a prostitute by their adult children and children-in-law, “*They won’t respect you. They’ll say do elder people also indulge themselves in prostitution? We thought only young ones. So where have the elderly contracted HIV from?*” (female, late fifties, stage 3). Those who had never been married were least protected from being accused of immoral behaviour: “*it’s no longer 50∶50*”.

However, women predominantly perceived treatment as a route to sustain their self-determination, “*this medication is my life and I cannot do without it*” (female, late thirties, stage 2). Many women sought and received regard for maintaining their care-giving roles, as mothers, sisters or wives, and their small businesses: “*I am not ashamed. I tell them openly I am on ARVs. I have a great responsibility to take care of my children and relatives so why should I shorten my life*” (female, mid-thirties, stage 2).

Men feared their friends, “*I was very much afraid that my friends would laugh at me, it takes courage to come here*” (male, late thirties, stage 3). The more authority men possessed the greater the fear of being laughed at, particularly by younger men. Adult men feared the loss of their right to respect from wives and relatives, “*People do not respect me. Even my wife does not respect me as before*” (male, late forties, stage 4); “*they say I am already finished*” (male, late forties, stage 3). Men expressed the shame of disclosing their status to their father or senior male relatives, “*I disclosed my status to my parents, especially my father. He said, ‘my son you have killed yourself’ so I just apologised to him*” (male, early forties, stage 4). However, early male attendees with resources such as educational attainments and formal employment derided other men's fears, “*men are stupid, they stay away without knowing they are killing themselves*” (male, mid-thirties, professional, stage 1). Among younger boys, obedience to parents and medical staff was paramount.

#### Responses to marital dissolution and reduced marriage chances

‘Being known’ to be HIV positive or on ART was perceived to reduce marriage chances. Male respondents found marriage essential “*my wife’s uncle refuses to allow her to come north. I want to marry another woman so she can assist me with housework and the problems I face at home when it comes to cooking*” (male, late thirties, stage 2).

Illness contributed to marital dissolution, “*because I was very sick at that time she ran away*” (male, early forties, stage 4); “*she told me, ‘I have gone back home because you are sick*’” (male, early thirties, stage 3), yet the desire for prompt remarriage discouraged men from attributing illness to HIV or seeking treatment. Of men presenting with advanced disease, two had remarried following the death of their wives but did not attribute the deaths to AIDS and two divorcees remarried in the year prior to testing and seeking treatment. Men who presented earlier for ART were less likely to have experienced recent marital breakdown or remarriage. Men's wives were frequently their treatment guardian.

Young women desired marriage and children: “yes, I *can get married, my blood is still flowing*” (female, late teens, stage 2); “*I long for a child*” (female, mid-teens, stage 1); though they acknowledged that marrying could pose problems for accessing treatment, “*It can be difficult to go together to the clinic*” (female, early thirties, stage 2). Widowhood or divorce, for reasons related or unrelated to HIV, could facilitate women's treatment seeking. Family members commonly intervened when a woman was sick, and her husband unsupportive, “*After I got sick, my husband stopped taking care of me. Then my brother came and took me away. He has instructed me not to go back*” (female, early thirties, Stage 3); “*When I explained my status to my husband he became violent...he refused to get tested…so I told my mother and she came for me”* (female, late twenties, stage 2). Women's relatives were almost always their treatment guardians. Particularly for older women, remarriage was less desirable than having boyfriends who could send assistance when requested. It was difficult to bring children to a new marital household, “*I can’t take the children to a new marriage. I have decided to stay at my home*” (female, early forties, stage 2).

### Supports to seeking care

#### Awareness of partner's positive HIV status

Disclosure of a positive HIV test result by a spouse encouraged men and women to seek treatment before severe health decline: “*I wasn’t worried about attending for treatment because my wife was already positive and this encouraged me*” (male, mid-forties, stage 2); “*I did not panic very much about the results because by then my husband was already on ARVs*” (female, early thirties, stage 1). Eight of the ten currently married women interviewed had husbands who were also receiving ART. Almost all married men screened before stage 4 had wives who had previously tested positive for HIV. Most partners assumed they were positive if their partner tested positive. Additionally, wives who had nursed sick or dying husbands were aware of the possible relationship to HIV. Three asymptomatic women (stage 1) described their husband's illness or death as the principal reason for seeking treatment, “*how he looked at that time made me go*” (late thirties, stage 1).

Some men still delayed. As one man's wife explained, “we came together but it was very hard to convince him in the first place...I remind him ‘Let’s go to the hospital, look you are losing weight’. He says ‘tomorrow’” (female, late thirties, stage 2). Wives who were afraid of disclosing a positive result waited until their husband showed signs of ill health, “my wife only told my sister. When I fell sick...my sister disclosed to me that my wife was already tested but they were afraid to tell me” (male, mid-thirties, stage 2). For men and women from polygynous unions, disclosure was additionally hampered by the burden of communicating results to co-wives.

#### Advice and support from relatives

Close relatives were an important source of confidential support. Their encouragement, alongside that of sympathetic spouses and friends, helped people overcome their fears of attending the clinic, “*if you’ve told a relative that you know very well and your wife, it becomes easier and you fear less*” (male, late thirties, stage 2). Both men and women were disadvantaged if they lacked close relatives, “*when I was told that I have the disease I said, ‘Where do I get help then?’ It became difficult for me to go and reveal this since my father was dead*” (male, early thirties, stage 3).

Women's natal relatives played a highly influential role, advising women to seek treatment, “*my parents are the ones who made me come here to the clinic*” (female, early thirties, stage 2). Women are generally nursed by a sister or their mother and return to their natal home in the event of serious or long-term illness. Relatives are therefore aware of any ill health. Women also know that they must seek treatment promptly if they are to continue to manage their own households without assistance. In contrast, men principally rely on their spouse for home nursing, and older men are more likely to refuse the advice of their relatives, preferring to dispense advice rather than seek it.

Disclosure helped women mobilise material support, including help with transport costs, food and housing, from their relatives, “*my mother and my brother are the ones that know my status and they are the ones that assist me. What’s the use of informing other people who will not assist me at all?*” (female, early thirties, stage 3). Men were less willing to compromise their independence and seek assistance if needed. Three men screened in stage 4 described ‘financial problems’ as a factor in their decision to delay treatment.

#### Responsibilities for children

Most respondents had children, although not all were co-resident. Responsibility for dependent children was described as an encouragement to seek and to stay on treatment by men and women. Men emphasised their role as provider: “*I tell them about my status so they realise school is important, ‘as our father is still alive he can help us go through education’ ”* (male, mid-thirties, stage 2); “*These days at least I work. It takes my wife to say ‘hoe down!’ I want to put iron sheets on my house so that as my child grows up he should live in a good house*” (male, late forties, stage 4). Women emphasised their role in children’s daily care and health, “*when a woman is sick it means all children experience a tough time at home. That is why we rush to the hospital*” (female, early forties, stage 2). Seeking assistance for children was an additional reason for women to disclose, and women used their maternal role to mitigate community HIV stigma. Children helped their mothers, reminding them to take their medicine, fetching the medication and water and providing comfort. One divorced man had his elder son returned to him by relatives, to provide him with help at home.

#### Quantitative Results

Since we expected different factors to be important for men and women, associations with late presentation (stage 3/4) were analysed separately by sex (table 2).

**Table 2:** Risk factors for late presentation for ART (stage 3/4 vs 1/2) by gender.

	Women						Men					
	n/N	%	OR	95% CI	aOR^a^	95% CI	n/N	%	OR	95% CI	aOR^a^	95% CI
Age												
15 – 29	14/47	29.8	0.74	0.35–1.6	0.34	0.12–0.98	6/12	50.0	1.1	0.31–3.8	0.87	0.17–4.4
30 – 39	34/98	36.6	1		1		25/52	48.1	1		1	
40 – 49	18/48	37.5	1.0	0.51–2.1	0.93	0.40–2.1	30/48	62.5	1.8	0.81–4.0	3.8	1.3–10.9
50 – 80	11/25	44.0	1.4	0.56–3.3	0.84	0.27–2.6	20/40	50.0	1.1	0.47–2.5	1.4	0.47–4.1
Area												
Remote	32/75	42.7	1		1		39/71	54.9	1		1	
Roadside	36/116	31.0	0.60	0.33–1.1	0.63	0.32–1.2	33/66	50.0	0.82	0.42–1.6	0.88	0.38–2.0
Marital Status												
Married	26/86	30.2	1		1		57/115	49.6	1		1	
Divorced	12/43	27.9	0.89	0.40–2.0	0.72	0.29–1.8	5/10	50.0	1.0	0.28–3.7	1.0	0.24–4.2
Widowed	20/45	44.4	1.8	0.87–3.9	1.6	0.69–3.6	1/2	50.0	1.0	0.06–16.7	0.58	0.03–11.1
Never Married	10/16	62.5	3.8	1.3–11.7	3.4	0.97–11.8	9/10	90.0	9.2	1.1–74.6	7.6	0.79–75.6
Education												
None/1–5 primary	15/37	40.5	1		1		11/18	61.1	1		1	
6–8 primary	36/99	36.4	0.84	0.39–1.8	0.98	0.39–2.4	37/68	54.4	0.76	0.26–2.2	1.0	0.29–3.5
Secondary or higher	17/54	31.5	0.67	0.28–1.6	0.80	0.29–2.21	23/50	46.0	0.54	0.18–1.6	0.81	0.19–3.4
Housing Quality												
Better Quality	30/87	34.5	1		1		32/67	47.8	1		1	
Poorer Quality	38/104	36.5	1.1	0.60–2.0	1.1	0.55–2.15	40/70	57.1	1.5	0.74–2.9	1.3	0.54–3.1
Dependent Children^b^												
None	27/67	40.3	1		1		39/56	69.6	1		1	
1–2	33/91	36.3	0.84	0.44–1.6	1.0	0.45–2.2	23/46	50.0	0.44	0.19–0.98	0.45	0.18–1.1
≥3	7/32	21.9	0.41	0.16–1.1	0.45	0.15–1.37	12/37	32.4	0.21	0.086–0.51	0.16	0.05–0.48

a adjusted OR's from multivariable model adjusted for age, area, marital status, education, housing quality and dependent, children.

b dependent children were defined as the number of biological children aged 14 years or below living in the household.

Among women, there was some evidence in the adjusted model that younger women (aged 15–29) were less likely to present in stage 3/4, and that never married women were more likely to present in stage 3/4 (adjusted OR 3.4, 95% CI 0.97–11.8). There was little variation by education level or housing quality, and only weak evidence that those with ≥3 dependent children presented earlier.

Among men, older men and never married men presented later. There was no evidence of associations with area of residence, education level or housing quality. Those with more dependent children presented earlier, and this persisted in the adjusted model.

Although there were differences between men and women in the cohort (men were older, more likely to be currently married, had higher level of schooling and more children), adjusting for these factors did not explain the association between gender and late presentation (adjusted OR 2.2, 95% CI1.3–3.9).

Since there was no evidence that the association between each of the investigated characteristics and late presentation varied by gender the data were combined. The factors found to be associated with later presentation are shown in table 3. In the final model, male gender (adjusted OR 2.3, 95% CI1.3–3.9) and never being married (adjusted OR 4.1, 95% CI1.5–11.5) were risk factors for late presentation, whereas having ≥3 dependent children was associated with earlier presentation (adjusted OR 0.31, 95% CI0.15–0.63).

**Table 3:** Factors associated with late presentation for ART Multivariable model.

	Patientsstage 3 and 4	Multivariable model	
Variables	n/N (%)	aOR (95% CI)^a^	P-value
Male gender	81/152 (53.3%)	2.26 (1.3–3.9)	**0.004**
Age			
15 – 29	20/59 (33.9)	0.48 (0.21–1.1)	0.09
30 – 39	59/145 (40.7)	1	
40 – 49	48/96 (50.0)	1.7 (0.91–3.0)	0.1
50–80	31/65 (47.7)	0.97(0.49–1.94)	0.9
Marital Status			
Married	83/201 (41.3)	1	
Divorced	17/53 (32.1)	0.78 (0.38–1.61)	0.5
Widowed	21/47 (44.7)	1.4 (0.65–3.0)	0.4
Never Married	19/26 (73.1)	4.1 (1.5–11.5)	**0.006**
Dependent Children^b^			
None	66/123 (53.7)	1	
1–2	56/137 (40.9)	0.76 (0.43–1.3)	0.33
3 or more	19/69 (27.5)	0.31 (0.15–0.63)	**0.001**

a OR's from final multivariable model adjusted for gender, age, marital status, children.

b dependent children were defined as the number of biological children aged 14 years or below living in the household.

The clinic attendees included 30 marital unions. The 39 spouses who were the second partner to attend, or who attended together, had less advanced disease than other married individuals (p = 0.004, Fisher's exact test). None were in stage 4 and one third were asymptomatic (stage 1) compared with 14% in stage 1 and 14% in stage 4 for those who were the first to attend. Men and women were equally likely to be the second partner to attend.

## Discussion

Among those presenting for ART, men had more advanced HIV-disease than did women. Only 6% of women presented in clinical stage 4 compared with 17% of men. Most men presenting with advanced disease described ignoring health promotion messages, had experienced ill health before testing and had postponed seeking treatment. Men did not want to display weakness nor invite the social consequences of ‘being known’ to be HIV positive earlier than they perceived to be necessary. Men feared the erosion of their right to respect from younger men, relatives, and wives. We also showed that men's need for (re)marriage at all ages may be a barrier to early treatment-seeking. Men's pattern of swift remarriage appears to be maintained in the face of HIV-related illness or widowhood [35].

Social position coupled with faster disease progression, may put older men, if not also women, at increased risk of late presentation. Older men (40–49 yrs) were more likely to present late and young women were more likely to present early (15–29 yrs) in the quantitative analysis. Never having been married was independently associated with late presentation, which was consistent with the qualitative finding that those who had never married were not protected from HIV stigma, more likely to desire marriage and less likely to be aware of partners' HIV status. We found no association between socio-economic indicators or distance from the road and late presentation, though the qualitative findings suggest it may be less appropriate to use household level socio-economic indicators than a measure which accounts for individuals' networks of assistance.

Women's defence against perceived shame focused on their commitment and responsibilities to children and relatives. Women's strong links with their natal family and long visits to natal homes provided a supportive structure which facilitated women's access to treatment, as relatives provided advice, assistance and enabled exit from unsupportive marriages. Women may use divorce to respond to perceived HIV risk in Malawi [36], complementing our finding that marital dissolution may facilitate women's access to treatment in the event of illness or ill treatment. More frequent testing, attention to descriptions of symptoms in health education, and close monitoring of their own and their partner's health, all improved women's awareness of their status before severe health decline. Widowhood however, did not necessarily lead to earlier disease-stage at presentation as shown in the quantitative analysis.

Men's main facilitating factors were having dependent children and the support of close relatives and a spouse. However, the collective effort of others to encourage men to seek treatment was hampered by their reluctance, which only waned after health decline. A plausible explanation for the strong quantitative association between increasing number of co-resident, dependent children and earlier treatment seeking in men is that this is a marker of marital stability as much as increased parental responsibility.

All patients must be accompanied by a friend or relative when they present for ART who is registered as their treatment guardian. Therefore some measure of social support and disclosure is essential to begin the treatment process. Married men and women described disclosure and support from a spouse also receiving or screening for ART as important, which was borne out by the quantitative analysis of this subgroup: in couples, those presenting second, or together, had less advanced disease than those presenting first.

Overall, this study suggests that people tend to wait for various forms of confirmation before taking the decision to seek treatment, from repeat testing to experiencing symptoms. Over half of men and a third of women in the cohort still had fairly advanced disease at screening, even if the proportion of women in stage 4 was low. Our findings that male gender, no children and never having been married were associated with late presentation are consistent with another rural African study [7]. The gendering of AIDS stigma and the perceived consequences of seeking treatment may also be relevant to other similar settings.

### Methodological Limitations

Since it was based in an ART clinic this study only included those who eventually decided to present for screening. Verbal autopsy work in the same population suggests men are also more likely than women to die without seeking ART at all; men dying of illnesses consistent with AIDS were less likely to have already been referred or on ART, than women dying of similar illnesses (47% compared to 67%, chi squared 5.06 p = 0.024, n = 127. 2007–2010 unpublished data). Transport costs were not identified as a major contributor to delays but could still be a factor in non-presentation among those dying without treatment. Another limitation was the relatively small quantitative dataset, although this was offset by the detailed data available on individuals within this cohort from the qualitative component. Since individuals were sampled retrospectively for interviewing, some of those with the most advanced disease had already died by the time of the study, particularly men. Since recruitment was clinic-based, respondents may have tended to emphasise negative community factors over negative health system factors.

The qualitative study informed the multivariable analysis however we were limited by the available data, and proxy variables give only an indication of whether the social processes described in the qualitative study occur more widely. Despite this, combining methods is extremely valuable in increasing understanding of the effect of the reported barriers and facilitators to accessing care and for exploring multiple social processes that may underlie single associations.

#### Implications

Our results emphasise the value of exploring as well as identifying behavioural correlates of late presentation. Efforts to enrol men into care before severe health decline should not only focus on physical health risks but the social consequences of debilitating conditions affecting work, family, relationships, and children. Perceived social risks often trump medical priorities [37]. The benefits of ART for sustaining valued masculine traits should be explicitly highlighted among men and those who could provide encouragement to them, just as female respectability and social inclusion is currently maintained with respect to care-giving.

Pragmatic approaches to increase access to treatment should be developed, such as health service adaptations to guarantee privacy and reduce public disclosure, and waiving of the requirement for a treatment guardian. The latter may be particularly important in increasing access by never married men and women. The findings support expansion of couples counselling services, with strong linkage between testing and screening. Community interventions which encourage older men with community seniority to endorse earlier treatment seeking and improve men's support networks may be valuable. Future implementation of revised recommendations for earlier initiation of ART (WHO 2009), in settings comparable to this study, increase the importance of identifying specific strategies to encourage early presentation in diverse groups.

## Acknowledgments

We thank the participants who shared their experiences of seeking ART, the Government of the Republic of Malawi for their interest in this Project and the National Health Sciences Research Committee of Malawi for permission to publish the paper.

## References

[1] Libamba E, Makombe S, Mhango E, de Ascurra TO, Limbambala E (2006). Supervision, monitoring and evaluation of nationwide scale-up of antiretroviral therapy in Malawi.. Bulletin WHO.

[2] Ferradini L, Jeannin A, Pinoges L, Izopet J, Odhiambo D (2006). Scaling up of highly active antiretroviral therapy in a rural district of Malawi: an effectiveness assessment.. The Lancet.

[3] Zachariah R, Fitzgerald M, Massaquoi M, Pasulani O, Arnould L (2006). Risk factors for high early mortality in patients on antiretroviral treatment in a rural district of Malawi.. AIDS.

[4] Jahn A, Floyd S, Crampin AC, Mwaungulu F, Mvula H (2008). Population-level effect of HIV on adult mortality and early evidence of reversal after introduction of antiretroviral therapy in Malawi.. Lancet.

[5] Chen SC-C, Yu JK-L, Harries AD, Bong C-N, Kolola-Dzimadzi R (2008). Increased mortality of male adults with AIDS related to poor compliance to antiretroviral therapy in Malawi.. Tropical Medicine and International Health.

[6] Kigozi IM, Dobkin LM, Martin JN, Geng EH, Muyindike W (2009). Late-disease stage at presentation to an HIV clinic in the era of free antiretroviral therapy in Sub-Saharan Africa.. J Acquir Immune Defic Syndr.

[7] May M, Boulle A, Phiri S, Messou E, Myer L (2010). Prognosis of patients with HIV-1 infection starting antiretroviral therapy in sub-Saharan Africa: a collaborative analysis of scale-up programmes.. Lancet.

[8] Cornell M, Myer L, Kaplan R, Bekker L-G, Wood R (2009). The impact of gender and income on survival and retention in a South African antiretroviral therapy programme.. Tropical Medicine and International Health.

[9] Krentz HB, Auld MC, Gill MJ (2004). The high cost of medical care for patients who present late (CD4 <200 cells/microL) with HIV infection.. HIV Medicine.

[10] Sabin CA, Smith CJ, Gumley H, Murphy G, Lampe FC (2004). Late presenters in the era of highly active anitretroviral therapy: uptake of and responses to antiretroviral therapy.. AIDS.

[11] Colebunders R, Ronald A, Katabira E, Sande M (2005). Rolling out antiretrovirals in Africa: there are still challenges ahead.. Clinical Infectious Diseases.

[12] Lima VD, Johnston K, Hogg RS, Levy AR, Harrigan PR (2008). Expanded access to highly active antiretroviral therapy: a potentially powerful strategy to curb the growth of the HIV epidemic.. J Infect Dis.

[13] Wringe A, Roura M, Urassa M, Busza J, Athanas V (2009). Doubts, denial and divine intervention: understanding delayed attendance and poor retention rates at a HIV treatment programme in rural Tanzania.. Aids Care.

[14] Grant E, Logie D, Masura M, Gorman D, Murray S (2009). Factors facilitating and challenging access and adherence to antiretroviral therapy in a township in the Zambian Copperbelt: a qualitative study.. AIDS Care.

[15] Murray L, Semrau K, McCurley E, Thea D, Scott N (2009). Barriers to acceptance and adherence of antiretroviral therapy in urban Zambian women: a qualitative study.. AIDS Care.

[16] Nyanzi-Wakholi B, Lara AM, Watera C, Munderi P, Gilks C (2009). The role of HIV testing, counselling, and treatment in coping with HIV/AIDS in Uganda: a qualitative analysis.. Aids Care.

[17] Unge C, Johansson A, Zachariah R, Some D, Engelgem I (2008). Reasons for unsatisfactory acceptance of antiretroviral therapy in the urban Kibera slum, Kenya.. AIDS Care.

[18] Kipp W, Alibhai A, Duncan Saunders L, Senthilselvan A, Kaler A (2010). Gender differences in antiretroviral treatment outcomes of HIV patients in rural Uganda.. AIDS Care.

[19] Nattrass N (2008). Gender and Access to Antiretroviral Treatment in South Africa.. Feminist Economics.

[20] Bila B, Egrot M (2009). Gender asymmetry in healthcare-facility attendance of people living with HIV/AIDS in Burkina Faso.. Social Science and Medicine.

[21] Fitzgerald M, Collumbien M, Hosegood V (2010). "No one can ask me 'Why do you take that stuff?'": men's experiences of antiretroviral treatment in South Africa.. Aids Care.

[22] Beck D (2004). Men and ARVs: How does being a man affect access to antiretroviral therapy in South Africa?.

[23] Seeley JA, Allison EH (2005). HIV/AIDS in fishing communities: challenges to delivering antiretroviral therapy to vulnerable groups.. Aids Care.

[24] Obermeyer CM, Sankara A, Bastien V, Parsons M (2009). Gender and HIV testing in Burkina Faso: an exploratory study.. Social Science and Medicine.

[25] Courtenay W (2000). Constructions of masculinity and their influence on men's well-being: a theory of gender and health.. Social Science and Medicine.

[26] Inhorn MC, Whittle LK (2001). Feminism meets the "new" epidemiologies: toward an appraisal of antifeminist biases in epidemiological research on women's health.. Social Science and Medicine.

[27] Hirsch J (2007). Gender, sexuality, and antiretroviral therapy: using social science to enhance outcomes and inform secondary prevention strategies.. AIDS.

[28] Dube A, Banda E, Msoma A, Floyd S, Molesworth A (2009). Fertility pattern in Karonga HDSS..

[29] Glynn JR, Kayuni N, Floyd S, Banda E, Francis-Chizororo M (2010). Age at menarche, schooling, and sexual debut in northern Malawi.. PLoS One.

[30] Marston M, Slaymaker E, Cremin I, Floyd S, McGrath N (2009). Trends in marriage and time spent single in sub-Saharan Africa: a comparative analysis of six population-based cohort studies and nine Demographic and Health Surveys.. Sex Transm Infect.

[31] Hemmings J (2007). Infertility and Women's Life Courses in Northern Malawi..

[32] Jahn A, Crampin AC, Glynn JR, Mwinuka V, Mwaiyeghele E (2007). Evaluation of a village-informant driven demographic surveillance system in Karonga, Northern Malawi.. Demographic Research.

[33] McGrath N, Kranzer K, Saul J, Crampin AC, Malema S (2007). Estimating the need for antiretroviral treatment and an assessment of a simplified HIV/AIDS case definition in rural Malawi.. AIDS.

[34] Morse JM, Tashakkori A, Teddlie C (2010). Procedures and Practice of Mixed Method Design: Maintaining Control, Rigor, and Complexity.. Sage Handbook of Mixed Methods in Social & Behavioural Research 2nd ed.

[35] Floyd S, Crampin AC, Glynn JR, Mwenebabu M, Mnkhondia S (2008). The long-term social and economic impact of HIV on the spouses of infected individuals in northern Malawi.. Tropical Medicine and International Health.

[36] Reniers G (2008). Marital strategies for regulating exposure to HIV.. Demography.

[37] Smith DJ, Mbakwem BC (2010). Antiretroviral therapy and reproductive life projects: mitigating the stigma of AIDS in Nigeria.. Social Science & Medicine.

